# Supplementary material for: A Quadruple Gene-Deleted Live BoHV-1 Subunit RVFV Vaccine Vector Reactivates from Latency and Replicates in the TG Neurons of Calves but Is Not Transported to and Shed from Nasal Mucosa
Source: Viruses. 2024 Sep 21;16(9):1497. doi: 10.3390/v16091497 (PMC11437494; doi:10.3390/v16091497)
Supplement: Supplementary file 1 [file viruses-16-01497-s001.zip › viruses-3180006-supplementary.pdf]

Supplementary Table S1: Clinical score in calves following Bovine herpesvirus type 1 (BoHV-1) infection and latency-reactivation. Dex. – dexamethasone; dp-Dex – day-post dexamethasone injection. Criteria: Rectal temperatures were scored 0–4 (<39.0 ◦C, 39.5 ◦C, 40.0 ◦C, 40.5 ◦C, >40.9 ◦C), nasal discharges were scored 0–4 (normal, serous, mild and severe mucopurulent), nasal lesions were scored 0–3 (normal, hyperemia, pustules and ulcers).

Group	Calf	Clinical score in calves. Days post-infection (Days post-Dex. injection)										
		Day 0	Day 3	Day 5	Day 7	Day 10	Day 14	Day 21	Day 28 (Day 0 dp-Dex)	Day 31 (Day 3 dp-Dex)	Day 33 (Day 5 dp-Dex)	Latency/ Reactivated
BoHV-1 wild-type	WT1-LAT	0	3	7	7	3	0	0	0	0	0	Latency
	WT2-LAT	1	2	6	6	3	0	0	1	0	0	
	WT3-RE	0	2	6	7	2	0	0	0	0	0	Reactivated
	WT4-RE	0	4	5	7	3	0	0	0	1	0	
	WT5-RE	0	3	7	6	3	0	0	0	0	0	
	**Average**	**0.2**	**2.8**	**6.2**	**6.6**	**2.8**	**0**	**0**	**0.2**	**0.2**	**0**	
BoHV-1qmv vector	VEC1-LAT	0	0	0	0	0	0	0	0	Euthanized	Euthanized	Latency
	VEC2-RE	0	0	0	0	0	0	0	0	0	1	Reactivated
	VEC3-RE	1	0	0	0	0	0	0	0	1	1	
	**Average**	**0.33**	**0**	**0**	**0**	**0**	**0**	**0**	**0**	**0.5**	**1**	
BoHV-1qmv Sub-RVFV vaccine	VAC1-LAT	0	0	0	0	0	1	0	0	Euthanized	Euthanized	Latency
	VAC2-LAT	0	0	0	0	0	0	0	0	Euthanized	Euthanized	
	VAC3-LAT	0	0	0	0	0	0	0	0	Euthanized	Euthanized	
	VAC4-LAT	0	0	0	0	0	0	0	0	Euthanized	Euthanized	
	VAC5-RE	0	0	0	0	0	0	0	0	0	1	Reactivated
	VAC6-RE	1	0	0	0	0	0	0	1	0	1	
	VAC7-RE	2	0	0	1	0	0	0	0	0	1	
	VAC8-RE	0	0	0	0	0	0	0	0	0	1	
	**Average**	**0.375**	**0**	**0**	**0.13**	**0**	**0.13**	**0**	**0.13**	**0**	**1**	

Supplementary Table S2. Nasal virus shedding following infection and dexamethasone-induced latency reactivation determined by the virus isolation in MDBK cells. ND – not determined. LAT-latency control; RE-Latency-reactivation.

Group	Animal #	Nasal shedding of BoHV-1 in PFU/ml of nasal swab. Day post-infection (Day post-Dexamethasone treatment)									
		Day 0	Day 3	Day 5	Day 14	Day 21	Day 26	Day 28 (Day 0)	Day 31 (Day 5)	Day 33 (Day 5)	Latency/ Reactivated
BoHV-1 wild-type	**WT1-LAT**	0	8.1x10^{4}	1.3x10^{5}	5.0x10^{1}	0	0	0	0	0	Latency
	**WT2-LAT**	0	6.3x10^{4}	2.6x10^{6}	0	0	0	0	0	0	
	**WT3-RE**	0	1.4x10^{5}	5.0x10^{5}	0	0	0	0	0	8.44 x 10^{2}	Reactivated
	**WT4-RE**	0	3.6x10^{3}	1.5x10^{5}	1.0x10^{2}	0	0	0	0	6.35 x 10^{2}	
	**WT5-RE**	0	4.2x10^{4}	6.5x10^{5}	0	0	0	0	0	5.0 x 10^{1}	
	**Average**	**0**	**6.6x10^{4}**	**8.1x10^{5}**	**3.0x10^{1}**	**0**	**0**	**0**	**0**	**5.09 x 10^{2} (RE)**	
BoHV-1qmv vector	**VEC1-LAT**	0	3.0x10^{3}	2.5x10^{2}	0	0	0	0	0	0	Latency
	**VEC2-RE**	0	2.0x10^{2}	0	0	0	0	0	0	0	Reactivated
	**VEC3-RE**	0	2.0x10^{2}	0	0	0	0	0	0	0	
	**Average**	**0**	**1.1x10^{3}**	**8.3x10^{1}**	**0**	**0**	**0**	**0**	**0**	**0**	
BoHV-1qmv Sub-RVFV vaccine	**VAC1-LAT**	0	4.8x10^{3}	0	0	0	0	Euthanized	Euthanized	0	Latency
	**VAC2-LAT**	0	4.0x10^{2}	0	0	0	0	Euthanized	Euthanized	0	
	**VAC3-LAT**	0	5.0x10^{1}	5.0x10^{1}	0	0	0	Euthanized	Euthanized	0	
	**VAC4-LAT**	0	2.0x10^{2}	0	0	0	0	Euthanized	Euthanized	0	
	**VAC5-RE**	0	5.0x10^{2}	0	0	0	0	0	0	0	Reactivated
	**VAC6-RE**	0	3.0x10^{3}	0	0	0	0	0	0	0	
	**VAC7-RE**	0	9.0x10^{2}	0	0	0	0	0	0	0	
	**VAC8-RE**	0	4.3x10^{3}	0	0	0	0	0	0	0	
	**Average**	**0**	**1.8x10^{3}**	**6.26**	**0**	**0**	**0**	**0**	**0**	**0**	

- Day 3 – BoHV-1 wt vs BoHV-1qmv vector – **60-fold higher in wt**.
- Day 3 – BoHV-1 wt vs BoHV-1qmv Sub-RFVF – **36-fold higher in wt**.
- BoHV-1 wt – Day 3 and day 5 – **12-fold higher on day 5**.
- BoHV-1qmv vector - Day 3 and day 5 - **13-fold lesser on day 5**.
- BoHV-1qmv Sub-RFVF - Day 3 and day 5 - **287-fold lesser on day 5**.
- Day 5 – wt vs qmv vector - **9,759-fold higher in wt**.
- Day 5 – wt vs BoHV-1qmv Sub-RFVF - **129,392-fold higher in wt**.

Supplementary Table S3. Quantification of bovine herpesvirus type 1 (BoHV-1) genome copies in nasal swab samples collected from the infected calves by qPCR.

Group	Calf	Nasal virus shedding following infection (viral genome copies/nasal swab) Days post-infection (Days post-Dexamethasone treatment)									
		Day 0	Day 3	Day 5	Day 14	Day 21	Day 26	Day 28 (Day 0 dp-Dex)	Day 31 (Day 3 dp-Dex)	Day 33 (Day 5 dp-Dex)	Latency/ Reactivated
BoHV-1 wild-type	WT1-LAT	0	127,740	194,700	185	0	0	0	0	0	Latency
	WT2-LAT	0	95,811	4593,230	0	0	0	0	0	0	
	WT3-RE	0	238,761	968,680	0	0	0	0	0	5436	Reactivated
	WT4-RE	0	54,423	232,000	793	0	0	0	0	8522	
	WT5-RE	0	66,832	109,700	0	0	0	0	0	488	
	**Average**	**0**	**116,713**	**1219,662**	**196**	**0**	**0**	**0**	**0**	**4815 (reactivated)**	
BoHV-1qmv vector	VEC1-LAT	0	138,585	144,680	0	0	0	0	0	Euthanized	Latency
	VEC2-RE	0	1900	1015	0	0	0	0	0	0	Reactivated
	VEC3-RE	0	17,694	1541	0	0	0	0	0	0	
	**Average**	**0**	**52,726**	**49,079**	**0**	**0**	**0**	**0**	**0**	**0**	
BoHV-1qmv Sub-RVFV vaccine	VAC1-LAT	0	241,080	10,871	0	0	0	0	Euthanized	Euthanized	Latency
	VAC2-LAT	0	5913	111,590	0	0	0	0	Euthanized	Euthanized	
	VAC3-LAT	0	22,754	27,315	0	0	0	0	Euthanized	Euthanized	
	VAC4-LAT	0	35,225	5859	0	0	0	0	Euthanized	Euthanized	
	VAC5-RE	0	10,032	13,810	0	0	0	0	0	0	Reactivated
	VAC6-RE	0	2868	945	0	0	0	0	0	0	
	VAC7-RE	0	5673	200,640	0	0	0	0	0	0	
	VAC8-RE	0	48,049	12,677	0	0	0	0	0	0	
	**Average**	**0**	**46,449**	**47,963**	**0**	**0**	**0**	**0**	**0**	**0**	

Supplementary Table S4: Quantifying targeted bovine herpesvirus type 1 (BoHV-1) genome (major capsid protein) and targeted transcript copies (ICP0, latency-related transcript, and glycoprotein C) in the trigeminal ganglion (TG) of infected/immunized calves following infection, latency, and reactivation.

Group	Calf	BoHV-1 genome and targeted transcript copies				
		BoHV-1 genome copies (per 10^6 cells)	ICP0 transcript copies (per ng of RNA)	Latency-related transcript copies (per ng of RNA)	Glycoprotein C transcript copies (per ng of RNA)	Latency/ Reactivated
BoHV-1 wild-type	WT1-LAT	7556	0	3212	0	Latency
	WT2-LAT	11,932	0	677	0	
	Average	**9744**	**0**	**1945**	**0**	
	WT3-RE	134,421	2440	0	87,134	Reactivated
	WT4-RE	99,332	4480	0	15,433	
	WT5-RE	203,991	1042	38	38,585	
	Average	**145,915**	**2,654**	**13**	**47,051**	
BoHV-1qmv vector	VEC1-LAT	756	0	102	0	Latency
	VEC2-RE	9630	42	0	884	Reactivated
	VEC3-RE	4946	111	0	439	
	Average	**7288**	**76.5**	**0**	**661.5**	
BoHV-1qmv Sub-RVFV vaccine	VAC1-LAT	997	0	32	0	Latency
	VAC2-LAT	273	0	28	0	
	VAC3-LAT	867	0	316	0	
	VAC4-LAT	1566	0	25	0	
	Average	**925.8**	**0**	**100**	**0**	
	VAC5-RE	7886	179	0	452	Reactivated
	VAC6-RE	11053	88	0	611	
	VAC7-RE	5739	141	0	984	
	VAC8-RE	7443	69	0	836	
	Average	**8030.3**	**119.3**	**0**	**720.8**	

Supplementary Table S5. Bovine herpesvirus type 1 (BoHV-1)-specific neutralizing antibody titer in serum determined by plaque-reduction assay.

Group	Calf	BoHV-1-specific neutralizing antibody titer in serum. Days post-infection (Days post-Dexamethasone treatment)						
		Day 0	Day 7	Day 14	Day 21	Day 28 (Day 0 dp-Dex)	Day 33 (Day 5 dp-Dex)	Latency/ Reactivated
BoHV-1 wild-type	WT1-LAT	<4	ND	ND	ND	88.49	82.29	Latency
	WT2-LAT	<4	ND	ND	ND	141.69	118.74	
	**Average**	**<4**	**ND**	**ND**	**ND**	**115.9**	**100.52**	
	WT3-RE	<4	ND	ND	ND	47.72	566.17	Reactivated
	WT4-RE	<4	ND	ND	ND	89.28	1281.55	
	WT5-RE	<4	ND	ND	ND	73.23	932.18	
	**Average**	**<4**	**ND**	**ND**	**ND**	**70.08**	**926.63**	
BoHV-1qmv vector	VEC1-LAT	<4	17.57	46.11	83.05	66.64	Euthanized	Latency
	VEC2-RE	<4	11.59	39.27	53.66	20.92	16.40	Reactivated
	VEC3-RE	<4	15.78	43.39	55.17	26.6	20.97	
	**Average**	**<4**	**14.98**	**42.92**	**63.96**	**38.05**	**18.66**	
BoHV-1qmv Sub-RVFV vaccine	VAC1-LAT	<4	18.84	61.37	94.35	28.62	Euthanized	Latency
	VAC2-LAT	<4	<4	5.67	13.12	12.5	Euthanized	
	VAC3-LAT	<4	4.11	5.26	11.40	8.15	Euthanized	
	VAC4-LAT	<4	<4	6.02	11.07	10.04	Euthanized	
	**Average**	**<4**	**5.74**	**19.6**	**32.5**	**14.8**		
	VAC5-RE	<4	22.12	70.45	103.6	41.63	39.62	Reactivated
	VAC6-RE	<4	7.43	8.80	16.53	10.91	9.94	
	VAC7-RE	<4	11.63	20.96	25.01	15.44	10.19	
	VAC8-RE	<4	7.42	9.03	14.73	11.61	7.93	
	**Average**	**<4**	**12.15**	**27.31**	**39.97**	**19.90**	**16.92**	
